# Local Applications to the Upper Air Passages

**Published:** 1907-12-07

**Authors:** 


### Laryngology and Rhinology.

### LOCAL APPLICATIONS TO THE UPPER AIR PASSAGES.

Steam inhalations are chiefly used in the treatment of catarrh of the larynx, and also of the trachea and bronchi; they are on the whole of more value in cases of acute or sub-acute inflammation than in the chronic forms of disease. Inhalations are usually ordered once to three times a day; each inhalation should be continued for five to ten minutes, the patient breathing deeply, slowly, and easily. A certain tendency to nausea may be produced, therefore the treatment should be practised before or between meals, but never immediately after food. The inhaling should be done in a warm room, and the patient must remain there for half an hour afterwards, for a hot steam inhalation undoubtedly renders him more susceptible to "catching cold." Many forms of apparatus are in use; the most important point is that all tubes and orifices must be wide enough to allow of free respiration without effort. An ordinary jug answers very well, a quart jug being used to contain a pint of the hot solution, with a towel arranged around the top in the form of a cone or cylinder to fit the mouth and nose. If a special inhaler be used it should be large enough to contain plenty of fluid, or it will rapidly become cool: it should have a face-piece like an anæsthetist's mask, for many patients find it difficult to inhale freely from the usual tubular-shaped inhalers. The temperature of the inhalation is very important, and a thermometer should always be used, for damage may be done if the steam be too hot; 140° F. is the correct temperature for the fluid, except when powerful volatile substances like chloroform are employed, when a temperature of 100° F. is sufficient. In prescribing medicaments for inhalation it is usual to order them of such strength that one teaspoonful is added to a pint of the hot water. Friar's balsam, or compound tincture of benzoin, makes a most valuable sedative inhalation, and the essential oils are much used where a more stimulating action is desired: oleum pini sylvestris, oleum eucalypti, oleum cubebæ and creosote are among those most often employed. The latter are the more powerful, and the former have the milder effect. All such essential oils may be prescribed mixed with water, and held in suspension by the addition of light magnesium carbonate or kaolin in the proportion of ½ grain to each minim of the oil, thus:—

| | | |
|---|---|---|
| Oleum Pini sylvestris | ... ... ... | ♏xl. |
| Magnesii Carb. Levis | ... ... ... | gr.xx. |
| Aquam | ... ... ... ... ... ad | ℥j. |

Menthol and thymol must first be dissolved in alcohol.

| | | |
|---|---|---|
| Menthol | ... ... ... ... ... ... | gr.xvi. |
| Spiritus Vini Rect. | ... ... ... ... | ʒij. |
| Magnesii Carb. Levis | ... ... ... | gr.viij. |
| Aquam | ... ... ... ... ... ad | ℥j. |

Bearing these points in mind, any combination or proportions of these drugs may be prescribed.

But the application of hot steam is not always advisable, and it certainly carries with it an increased liability to "catching cold," especially if the patient is going about and doing his work. The medicaments can be introduced at the ordinary temperature in a finely divided state as a spray, and this method has become very popular with the recent improvement in apparatus. A moderately coarse spray is to be used to apply drugs to the nose and pharynx, but a very fine divided vapour reaches the larynx and trachea, and even penetrates to the smaller bronchi. The spray may be directed straight on to the pharynx through the mouth, but an oily medicament intended for the larynx will reach the affected part just as well if inhaled through the nose, and the unpleasant taste in the mouth will thus be avoided. The medium for the nebula may be oily or aqueous; for the former a light tasteless petroleum oil is in general use, such as the paraffinum liquidum of the British Pharmacopœia, though this has rather too high a specific gravity. The essential oils dissolve readily in this medium, and are usually employed in about the same quantities to the ounce as are prescribed for steam inhalations. It must be remembered, however, that neither alkaloids nor their salts dissolve readily in these hydrocarbon oils; if an alkaloid is to be used the basal form should be prescribed and some oleic acid added to dissolve it, thus:—

| | | |
|---|---|---|
| Menthol | ... ... ... ... ... ... | gr.xv. |
| Cocaine | ... ... ... ... ... ... | gr.v. |
| Oleic Acid. | ... ... ... ... ... ... | ♏xv. |
| Paraffinum Liquidum | ... ... ad | ℥j. |

This is merely an example of prescribing, and must not be taken to imply that the use of cocaine in a nebuliser is advisable except in special circumstances. Aqueous solutions are less often employed in the nebuliser, though a coarse aqueous spray is of use to apply astringents to the pharynx or as a substitute for the nasal syringe. Water alone will not make a fine nebula in the usual instruments; the addition of 20 to 25 per cent. of glycerine is necessary, and, in general, the thicker the fluid the finer is the resulting spray. Astringents and antiseptics may usefully be applied to the larynx in an aqueous nebula, and the following are examples of serviceable prescriptions:—

| | | |
|---|---|---|
| Iodi | .. ... ... ... ... | gr.vj. |
| Potassii Iodidi | ... ... ... ... ... | gr.xij. |
| Acidi Carbolici | ... ... ... ... ... | gr.x. |
| Glycerini | ... ... ... ... ... | ʒiss. |
| Aquam | ... ... ... ... ad | ℥j. |

| | | |
|---|---|---|
| Acidi Tannici | ... ... ... ... | grxx. |
| Glycerini | ... ... ... ... ... | ʒiss. |
| Aquam | ... ... ... ... ad | ℥j. |

As to apparatus, it is most convenient to use an instrument with a small nozzle which readily fits the nostril, and it is important that the hand-ball be large, and that a good volume of vapour be produced with little effort. The ordinary rubber ball acts well enough for most cases, but the requisite pressure can also be obtained by a mechanical pump or a compressed-air apparatus, or by steam, as in Siegle's excellent steam-spray. A larger apparatus is necessary when it is desired to treat pulmonary disease by inhalation, and at several health-resorts large mechanical vaporisers are in use for this purpose.

